# A Network Analysis of Post-traumatic Stress Disorder Symptoms and Correlates During the COVID-19 Pandemic

**DOI:** 10.3389/fpsyt.2020.568037

**Published:** 2020-11-10

**Authors:** Wanyue Jiang, Zhihong Ren, Lixia Yu, Yafei Tan, Congrong Shi

**Affiliations:** ^1^School of Psychology, Central China Normal University, Wuhan, China; ^2^Key Laboratory of Adolescent Cyberpsychology and Behavior, Ministry of Education, Wuhan, China; ^3^Key Laboratory of Human Development and Mental Health of Hubei Province, Wuhan, China

**Keywords:** network analysis, coronavirus disease 2019 (COVID-19), centrality, post-traumatic stress disorder (PTSD), suicide, public

## Abstract

**Background and Objective:** The coronavirus disease 2019 (COVID-19) outbreak has been suggested as a collective trauma, which presents a continuing crisis. However, the specific post-traumatic implication of this crisis has not been adequately studied yet. The current study was aimed to ascertain the most central symptom and the strong connections between symptoms of post-traumatic stress disorder (PTSD). At the same time, exploring the relationship between covariates and the network of PTSD symptoms, by taking sex, anxiety, depression, suicidal ideation, quality of life, and social support as covariates, may help us to know the arise and maintenance of PTSD symptoms and give specified suggestions to people under the shadow of COVID-19.

**Method:** The Post-traumatic Stress Disorder Checklist for Diagnostic and Statistical Manual of Mental Disorders, Fifth Edition (DSM-5), was used to assess the PTSD symptoms extent of 338 healthy participants over the past month. Networks were analyzed using state-of-the-art regularized partial correlation models. In addition, the centrality of the symptoms and the robustness of the results were analyzed.

**Results:** The network analysis revealed that the especially strong connections emerged between avoidance of thoughts and avoidance of reminders, hypervigilance and exaggerated startle response, intrusive thoughts and nightmares, flashbacks and emotional cue reactivity, and detachment and restricted affect. The most central symptoms were self-destructive/reckless behavior. Incorporation of covariates into the network revealed the strong connections path between self-destructive/reckless behavior and loss of interest and depression.

**Conclusion:** Self-destructive/reckless behavior was the most central symptom in the network of PTSD symptoms related to the COVID-19 pandemic, which as an important target of interfere may have great benefits.

## Introduction

Coronavirus disease 2019 (COVID-19) presents one of the greatest global public health threats of the twenty first century. On March 11, 2020, the World Health Organization (WHO) declared the COVID-19 coronavirus outbreak a pandemic. In China, the government advised citizens into home quarantine and inhibited most public transportations on January 23, 2020. COVID-19 has impacted every aspect of society (1). It has not only caused physical health issues, the pandemic and the need for isolation have also increased psychological health problems, including post-traumatic stress disorder (PTSD), depression, anxiety, and widespread fear (2, 3). Moreover, due to unprecedented levels of documentation and public exposure, COVID-19 may affect the majority of the population and cause vicarious trauma (4). The COVID-19 outbreak has been suggested as a collective trauma, which is a continuing crisis for everyone (5–7). However, the specific post-traumatic implication of this crisis has not been adequately studied yet. To prevent potential PTSD, it is necessary to investigate the characteristics of symptoms related to traumatic stress in people exposed to the COVID-19 crisis.

PTSD follows traumatic events and is characterized by symptoms of avoidance, intrusions, excessive arousal, and emotional numbing, etc (8). Previous studies on PTSD mostly adopted the reflective models based on the common cause hypothesis (9, 10). According to these models, symptoms reflect an underlying latent construct (i.e., disorder), which means the symptom covariance is caused by the latent construct, and it is causally independent among the symptoms themselves (11). For example, based on this perspective, studies on the prevalence of PTSD during COVID-19 found that about 10% of the population meet the PTSD criteria, and subthreshold disturbances accounted for a large proportion of PTSD disturbance (6, 12–15). Recently, McNally et al. (16) have proposed a causal system and suggested causal connections among PTSD symptoms that occur. For example, survivors who are exposed to trauma cues will likely be reactive and aroused, leading to avoidance behaviors. In addition, Ehlers and Clark (17) have assumed that individuals may have a negative bias in the evaluation of trauma and its outcomes after experiencing a traumatic event, and negative bias will cause avoidance of trauma cues, thereby increasing the sensitivity to threat and level of anxiety, leading to a vicious circle, which tend to maintain the PTSD symptoms. Empirical studies also showed that the factor structure of PTSD symptoms was varying in different traumatic experiences (18–20). Moreover, the relationships between PTSD symptoms and other psychological symptoms (e.g., anxiety, depression, and quality of life) (21–24), as well as the responses to treatment are changeable for different PTSD symptoms (25). However, neither the most central PTSD symptoms related to COVID-19 nor the related covariates were clear yet.

Network analysis has emerged as an approach involved in causal systems perspective. Specifically, network analysis is a methodology based on graph theory. Such methodology could be used to visualize the interaction between all observed variables, including psychopathology symptoms (26). The underlying hypothesis is that symptoms are interdependent, and a psychological disorder constitutes a network of symptoms that interact (11, 27, 28). Furthermore, network analysis enables computation of centrality that reveals the most important target of clinical interventions (9). Recently, network analysis has been applied to examine the constructs of mental disorders such as depression, schizophrenia, and anxiety disorders (29–32).

Network analysis has been used to identify the construct of PTSD, revealing that the factor structure of symptoms varied in different traumatic events. The studies have consistently found strong connections between hypervigilance and exaggerated startle response and between flashbacks and nightmares (16, 33–37). However, there is no agreement on the most central symptoms yet. The following symptoms have been identified as central symptoms of PTSD: negative trauma-related emotions (33), feeling emotionally numb (34), intrusions and concentration deficits (35), intrusive recollections and flashbacks (36), feeling detached (27), hypervigilance (16), and emotional cue reactivity (37). The researchers attributed the discrepancy to the different traumatic events, including natural disasters, wars, accidents (e.g., car accidents), man-made disasters (e.g., abuse), etc. However, a cross-cultural study showed moderate to high correlations of network structure and centrality estimates between four trauma patient samples with different cultures and types and severity of trauma (38). COVID-19 has been suggested as a new type of mass trauma (5) or a collective trauma (7), which was different from trauma on an individual level. It is necessary to investigate the PTSD symptom network related to the COVID-19 pandemic and further examine the most central symptoms so as to develop more targeted interventions.

In addition, previous studies have revealed individual difference in the network of PTSD symptoms. For example, Armor et al. (33) included sex, age, anxiety, depression, suicidal ideation, mental and physical functioning, and quality of life in the PTSD symptom network and found a strong connection between self-destructive/reckless behavior and suicidal ideation. They also found associations between difficulty concentrating and anxiety and depression, as well as associations between quality of life and restricted affect and depression. The findings suggested considering depression, anxiety, and suicidal ideation when diagnosing and treating PTSD. In order to expand the PTSD symptom network, Birkeland and Heir (34) included sex, severity of exposure, and social support as covariates. The results showed that women had a stronger physiological cue activity compared to men and a correlation between low social support and difficulty sleeping. Cao et al. (36) emphasized the impact of sex and revealed sex differences in both global connectivity and individual symptoms' connectivity of PTSD symptom networks. These findings indicate that females and persons who receive less social support are relatively more vulnerable to PTSD when they are exposed to traumatic events.

Conclusively, network analysis reveals the interactions among symptoms and the relationships between symptoms and covariates. It is still unclear for the PTSD symptom network and the relationships between covariates and symptoms of the people who were exposed to the COVID-19 outbreak. Therefore, the present study aimed to investigate the network of PTSD symptoms and the most central symptoms on populations who were exposed to the COVID-19 outbreak to examine the role of covariates including sex, anxiety, depression, suicidal ideation, social support, and quality of life in the PTSD symptom network.

## Methods

### Participants

This study was conducted between April 4 and April 10, 2020, when the government ended the lockdown of Wuhan and the COVID-19 crisis was under control in China (April 8). Questionnaires were distributed online using a snowball sampling approach. Specifically, we posted advertisements that described the purpose of the study and the principle of voluntary participation on well-known social software (WeChat and Tencent QQ) in China. The participants recruited in the study voluntarily shared the advertisements to relatives and friends. We used online questionnaires to collect data through the Questionnaire Star platform. A total of 361 questionnaires were completed. A total of 338 (252 females) valid questionnaires were analyzed after deleting recurring responses. The subjects were paid 3 yuan after completing the survey.

The average age of participants was 25.76 years (SD = 9.61). Among them, 19.8% of the participants were in Hubei Province, among which 48.5% of them were in Wuhan, the hardest-hit area in China during the COVID-19 outbreak. Moreover, 67.9% of the participants had a bachelor's degree, and 20.3% had a master's degree or above. Additionally, most of the participants were unmarried (81.3%). Also, 64.6% of the participants were students, 28.9% had a stable job, and 6.5% were unemployed. In addition, 16.7% of the participants worked as a volunteer during the COVID-19 outbreak, and 0.3% had been infected with COVID-19. The studies involving human participants were reviewed and approved by the Ethic Institutional Review Board of Central China Normal University.

### Measures

#### Post-traumatic Stress Disorder Symptoms

PTSD symptoms were assessed by the Post-traumatic Stress Disorder Checklist for Diagnostic and Statistical Manual of Mental Disorders, Fifth Edition (DSM-5) (PCL-5) (39, 40). The PCL-5 is a self-report measure and contains 20 items that correspond to the DSM-5 symptoms of PTSD. It measures the severity of PTSD symptoms over the last 1 month, rated on a 5-point Likert scale ranging from 0 (not at all) to 4 (extremely). To ensure that the PTSD symptoms we measured were related to the COVID-19 pandemic, we specified traumatic event as the COVID pandemic in the instructions. The Cronbach's alpha coefficient of PCL-5 was 0.94 in our study.

#### Depression and Anxiety Symptoms

Depression and anxiety symptoms were measured using Patient Health Questionnaire-4 (PHQ-4) (41), which is a self-assessment screening tool for depression and anxiety. The PHQ-4 consists of four items; the depression subscale includes two items and the anxiety subscale includes two items. The response options range from 0 (never) to 3 (nearly everyday). Each total score of the subscales indicates the severity of depression and anxiety, respectively, in which higher scores reflect greater severity of symptoms. In the present study, the Cronbach's alpha coefficient of depression subscale was 0.74, and the Cronbach's alpha coefficient of anxiety subscale was 0.81.

#### Suicidal Ideation

Suicidal ideation was assessed by the revised suicidal ideation subscale of PHQ-9 (42). The subscale contains two items, which evaluates passive and active suicidal ideation, respectively (43). Specifically, the items are “How often have you been bothered by the thoughts that you would be better off dead?” and “How often have you been bothered by the thoughts of hurting yourself?” over the last 2 weeks. The response options range from 0 (never) to 3 (nearly everyday). Higher summary scores indicate stronger suicidal ideation. The Cronbach's alpha coefficient of suicidal ideation subscale was 0.90 in our study.

#### Social Support

The Crisis Support Scale (CSS) (44) was used to measure the social support that the participants received during the COVID-19 outbreak. The CSS includes seven items that are answered on a 7-point Likert scale ranging from 1 (never) to 7 (always). Higher total scores reflect higher social support. The Cronbach's alpha coefficient of CSS was 0.82 in the present study.

#### Quality of Life

The quality of life was measured using the 12-item Short-Form Health Survey (SF-12) (45). The SF-12 has been widely used to evaluate the quality of life related to health, reflecting individual health status and impact of health status on daily life. The questionnaire contains two subscales including 12 items: physical health and mental health. The raw scores have been transformed into standard score (mean = 50, SD = 10) (46). The range of standardized score was 0 to 100. The quality of life was indicated by average score of the two subscales, and higher scores reflect better quality of life. In this study, the Cronbach's alpha coefficient of SF-12 was 0.80.

### Data Analysis

We used SPSS 24.0 (IBM Corp., Armonk, USA) to analyze participant characteristics. Estimations of network, centrality, and robustness were carried out in the free statistical environment R, following the suggestion of the developers on network analysis (47).

#### Network Estimate and Visualization

Two networks were estimated and visualized using R-package qgraph (48). We build a network containing 20 PTSD symptoms. In addition, we included six covariates (sex, anxiety, depression, suicidal ideation, social support, and quality of life) in the PTSD symptom network. The network consists of nodes and edges. In this present study, symptoms and covariates are “nodes,” and the relationships between the nodes are “edges.” We estimated the network of partial correlation coefficients *via* Gaussian Graphical Model. That is, the edge between two nodes was weighted connection controlling for all other edges in the network. It can be understood as a partial correlation, representing conditional independence associations, in which the range of the weight is from −1 to 1 (49).

Specifically, we estimated all the association parameters among the nodes of the network using the cor_auto of R package qgraph. It estimates a large number of parameters (i.e., 190 pairwise association parameters in the network with 20 nodes, 325 pairwise association parameters in the network with 26 nodes) that may result in some false-positive connections. To minimize the false-positive connections, we set small edges to zero by applying a regularization method (EBOCglasso) that was revised from the least absolute shrinkage and selection operator (50, 51). In addition, we calculated and visualized the networks using R package qgraph and bootnet. Nodes with stronger average associations were placed closer to the center of the graph via Fruchterman–Reingold algorithm (52). The green edges indicate positive associations, while the red edges represent negative associations. Furthermore, the thickness of the edges reflects the magnitude of the connection; that is, thicker edges indicate stronger connections.

#### Centrality Estimate

We calculated node centrality in the PTSD symptom network to identify the most central symptoms. Higher centrality indicates that the symptom has stronger connections with other symptoms (26, 47). For each node, we estimated three commonly used indices of centrality: strength, closeness, and betweenness (53). Strength was calculated as the sum of edge weights of a node, reflecting direct connection strength of a node with other nodes in the network. Closeness was indexed by the inverse of the sum of distance from the node to all other nodes, indicating indirect connection strength of a node with other nodes in the network. The path between one node and the other node is shorter, the influence of this node on the other one is greater. Betweenness was assessed as the frequency that a node lies on the shortest path between two nodes, which indicated how central the node was when connecting all other nodes in the network.

In addition, expected influence (EI) indicates centrality by estimating the sum of the original score of each node (54), which was involved with the weight of connections as well as the direction of connections (55). Exploratively, we estimated one-step EI using R package bootnet (47) and compared it with the centrality index above. Higher EI represents higher centrality of a node (27, 56, 57).

#### Robustness Estimation and Testing for Significance

Estimation of robustness (i.e., accuracy and stability) of a psychopathology network is still a main challenge in network analysis. As suggested by Epskamp et al. (47), we used R package bootnet to assess the robustness of networks in our study. Bootstrapping of R package bootnet was used to test the robustness of edge weights and the robustness of centrality indices.

First, we calculated 95% confidence intervals for the edge weights and tested for differences in edge weights and centrality indices based on 1,000 bootstrap iterations at the alpha level of 0.05. Second, a node-dropping subsetting bootstrap technique and the correlation stability (CS) coefficient were applied to estimate the stability of centrality indices. That is, if the correlation between centrality values calculated from a subsample with participants missing and centrality values calculated from the complete data set is high (>0.7 by default), we would consider that the centrality metric is stable. The CS coefficient is an index for centrality stability, and the value should be more than 0.25, preferably higher than 0.5 (47).

We estimated the robustness of edge weights in both the PTSD symptom network and the network with covariates, while we only assessed the stability of centrality indices in the PTSD symptom network.

## Results

### Sample Characteristics

The mean PCL-5 score was 12.90 (SD = 11.07). Also, 3.5% of the 338 participants reported a sum of PTSD symptoms over the PCL-5 cut point at 38 (58), 25.44% fulfilled two or more than two criteria of the B-E diagnosis criteria but with total PCL-5 scores of under 38 (59). Table 1 shows the means and standard deviations (SDs) of PTSD symptoms and covariates.

**Table 1:** Means and standard deviations of post-traumatic stress disorder (PTSD) symptoms and covariates.

	**Mean**	**SD**
**INTRUSIONS**		
B1: Intrusive thoughts	0.88	0.84
B2: Nightmares	0.38	0.69
B3: Flashbacks	0.71	0.87
B4: Emotional cue reactivity	0.87	0.83
B5: Physiological cue reactivity	0.45	0.69
**AVOIDANCE**		
C1: Avoidance of thoughts	0.67	0.86
C2: Avoidance of reminders	0.55	0.79
**COGNITION AND MOOD ALTERATIONS**		
D1: Trauma-related amnesia	0.57	0.80
D2: Negative belief	0.54	0.80
D3: Blame of self or others;	0.62	0.77
D4: Negative trauma-related emotions	0.70	0.79
D5: Loss of interest	0.68	0.88
D6: Detachment	0.84	0.96
D7: Restricted affect	0.60	0.84
**AROUSAL AND REACTIVITY ALTERATIONS**		
E1: Irritability	0.73	0.91
E2: Self-destructive/reckless behavior	0.18	0.58
E3: Hypervigilance	0.68	0.86
E4: Exaggerated startle response	0.59	0.78
E5: Difficulty concentrating	0.96	0.96
E6: Sleep disturbance	0.69	0.87
**COVARIATES**		
Anxiety	1.14	1.28
Depression	1.09	1.17
Suicidal ideation	0.29	0.88
Social support	30.94	7.96
Quality of life	49.33	6.12

### Post-traumatic Stress Disorder Symptom Network

**Figure 1:**
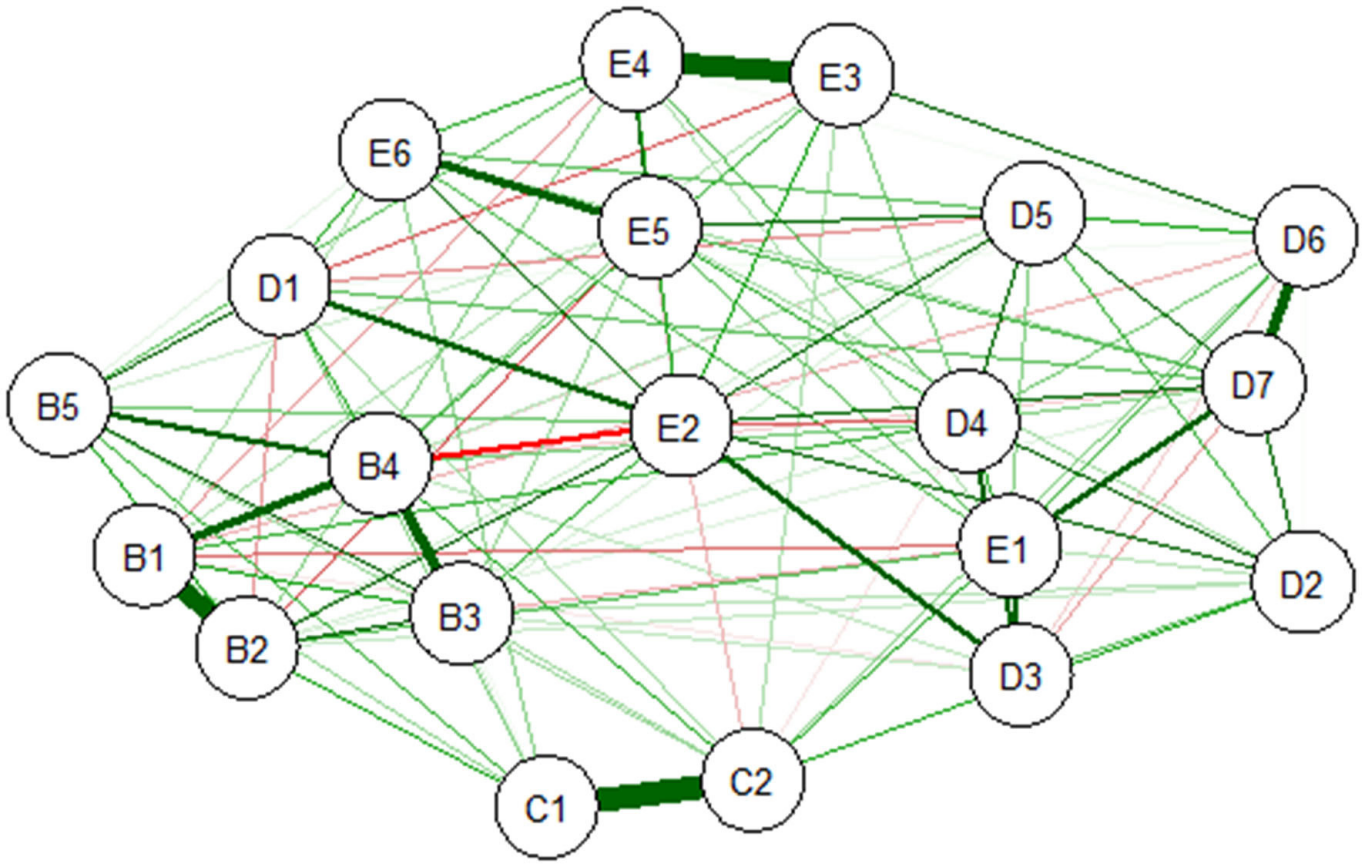
Estimated network of DSM-5 PTSD symptoms. B1, Intrusive thoughts; B2, Nightmares; B3, Flashbacks; B4, Emotional cue reactivity; B5, Physiological cue reactivity; C1, Avoidance of thoughts; C2, Avoidance of reminders; D1, Trauma-related amnesia; D2, Negative belief; D3, Blame of self or others; D4, Negative trauma-related emotions; D5, Loss of interest; D6, Detachment; D7, Restricted affect; E1, Irritability; E2, Self-destructive/reckless behavior; E3, Hypervigilance; E4, Exaggerated startle response; E5, Difficulty concentrating; E6, Sleep disturbance; PTSD, post-traumatic stress disorder; DSM-5, Diagnostic and Statistical Manual of Mental Disorders, Fifth Edition.

Figure 1 shows the network structure of the 20 PTSD symptoms. Most of the connections between symptoms were positive. The bootstrap difference test indicated five associations significantly higher than at least half of the other edges: between avoidance of thoughts (C1) and avoidance of reminders (C2), hypervigilance (E3) and exaggerated startle response (E4), intrusive thoughts (B1) and nightmares (B2), flashbacks (B3) and emotional cue reactivity (B4), and detachment (D6) and restricted affect (D7) (shown in Supplementary Figure 1 in Supplemental Material).

**Figure 2:**
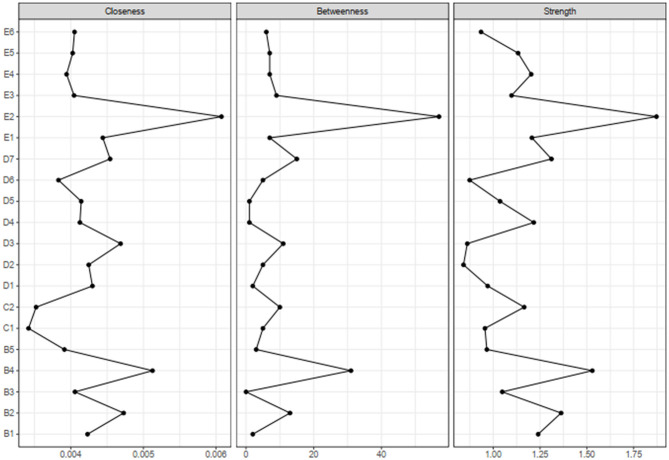
Centrality indices for the estimated network of DSM-5 PTSD symptoms. PTSD, post-traumatic stress disorder; DSM-5, Diagnostic and Statistical Manual of Mental Disorders, Fifth Edition.

The centrality indices (strength, closeness, and betweenness) are shown in Figure 2. The three indices were significantly intercorrelated with each other (the correlation between strength and closeness was 0.59 (*p* < 0.01), the correlation between strength and betweenness was 0.72 (*p* < 0.01), and the correlation between closeness and betweenness was 0.81 (*p* < 0.01). Recent studies have suggested that betweenness and closeness were unstable (56, 57). Thus, we only focused on strength because of its reliability and the high correlations with other indices. The results showed that five symptoms [Self-destructive/reckless behavior (E2), Emotional cue reactivity (B4), Nightmares (B2), Restricted affect (D7), and Intrusive thoughts (B1)] had a high node strength. Significance testing indicated that only strength for Self-destructive/reckless behavior (E2) was significantly higher than other nodes (shown in Supplementary Figure 2 in Supplemental Material). Trauma-related amnesia (D1) and blame of self or others (D3) showed a relatively lower node strength.

Additionally, the results showed that EI was significantly correlated with strength (*r* = 0.80, *p* < 0.01). EI analysis revealed that the restricted affect (D7), Self-destructive/reckless behavior (E2), exaggerated startle response (E4), nightmares (B2), and avoidance of reminders (C2) were significantly intercorrelated with each other (Supplementary Figure 3).

### Post-traumatic Stress Disorder Network With Covariates

**Figure 3:**
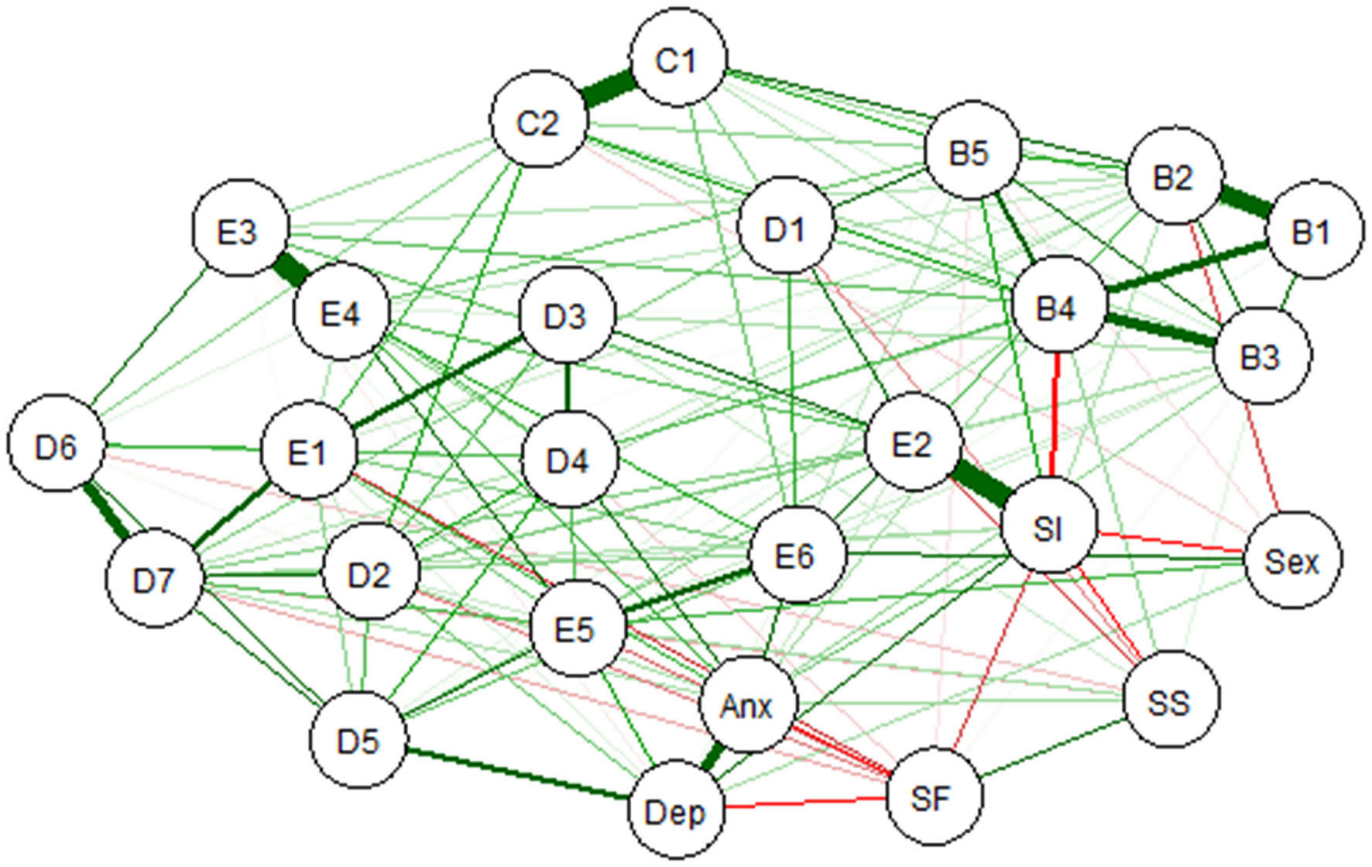
Estimated network of DSM-5 PTSD symptoms including covariates. Anx, anxiety; Dep, depression; SI, suicidal ideation; SS, social support; SF, quality of life; PTSD, post-traumatic stress disorder; DSM-5, Diagnostic and Statistical Manual of Mental Disorders, Fifth Edition.

Figure 3 shows the network of PTSD symptoms including six covariates, namely, sex, anxiety, depression, suicidal ideation, social support, and quality of life. The results indicated strong connections between self-destructive/reckless behavior (E2) and suicidal ideation (0.83) and between loss of interest (D5) and depression (0.66). In addition, anxiety and depression were positively correlated (0.73), and suicidal ideation and quality of life were negatively associated (−0.61).

### Robustness of Networks

**Figure 4:**
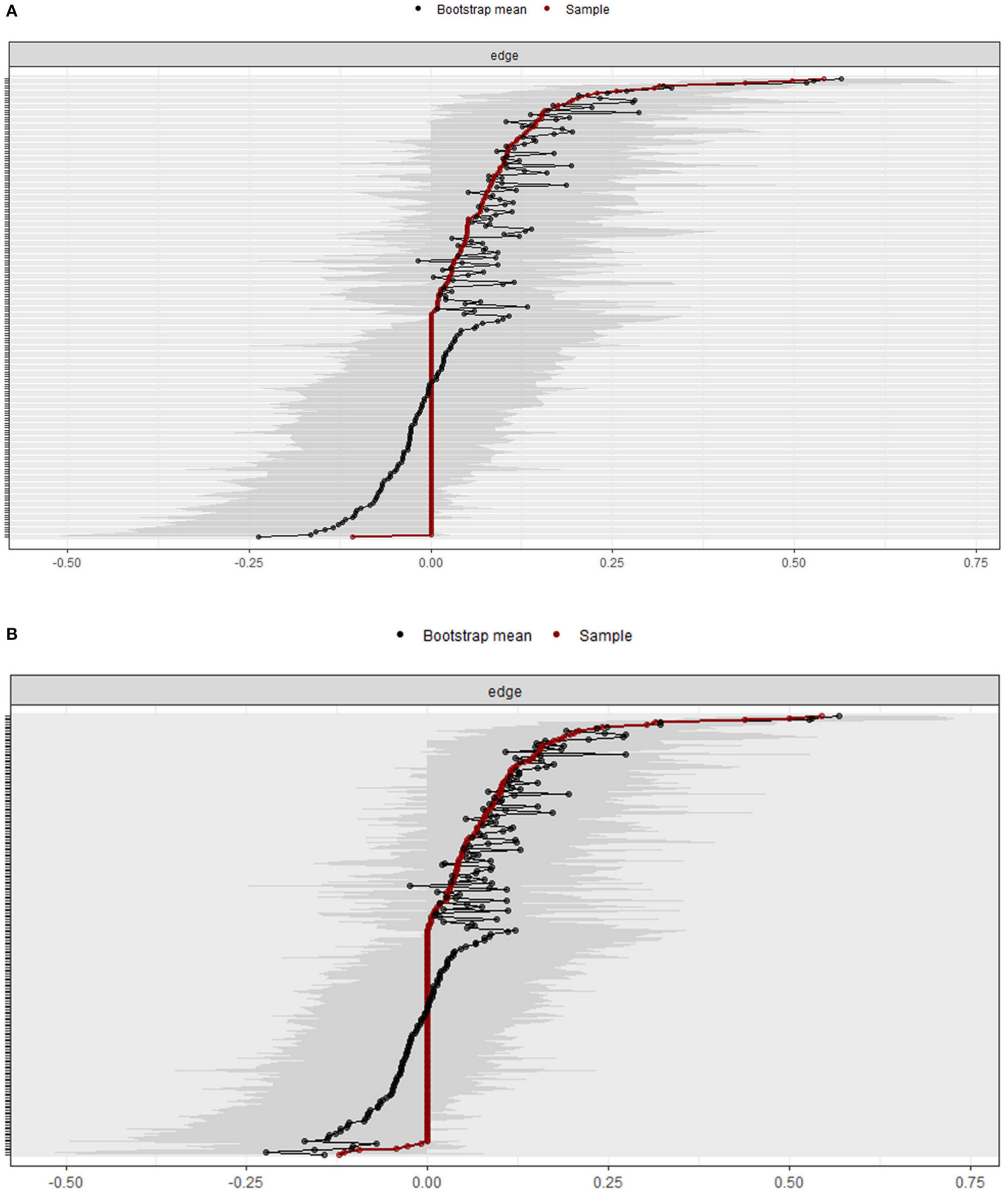
Robustness of networks. **(A)** Bootstrap 95% confidence intervals for estimated edge weights in 20 PTSD symptom network. **(B)** Bootstrap 95% confidence intervals for estimated edge weights in PTSD symptom network with covariates. Red line presents the edge weights. The 95% confidence intervals are presented by the gray area. PTSD, post-traumatic stress disorder.

The estimated robustness (i.e., stability and accuracy) of 20 PTSD symptom network was presented in Figure 4A. The estimated robustness of PTSD symptom network with covariates (26 nodes) was shown in Figure 4B. The results showed that 95% confidence intervals for the edge weights were mostly overlapping in both networks. The bootstrap testing for the edge weights indicated that the estimation of the PTSD symptom network and the significance were accurate in both networks.

**Figure 5:**
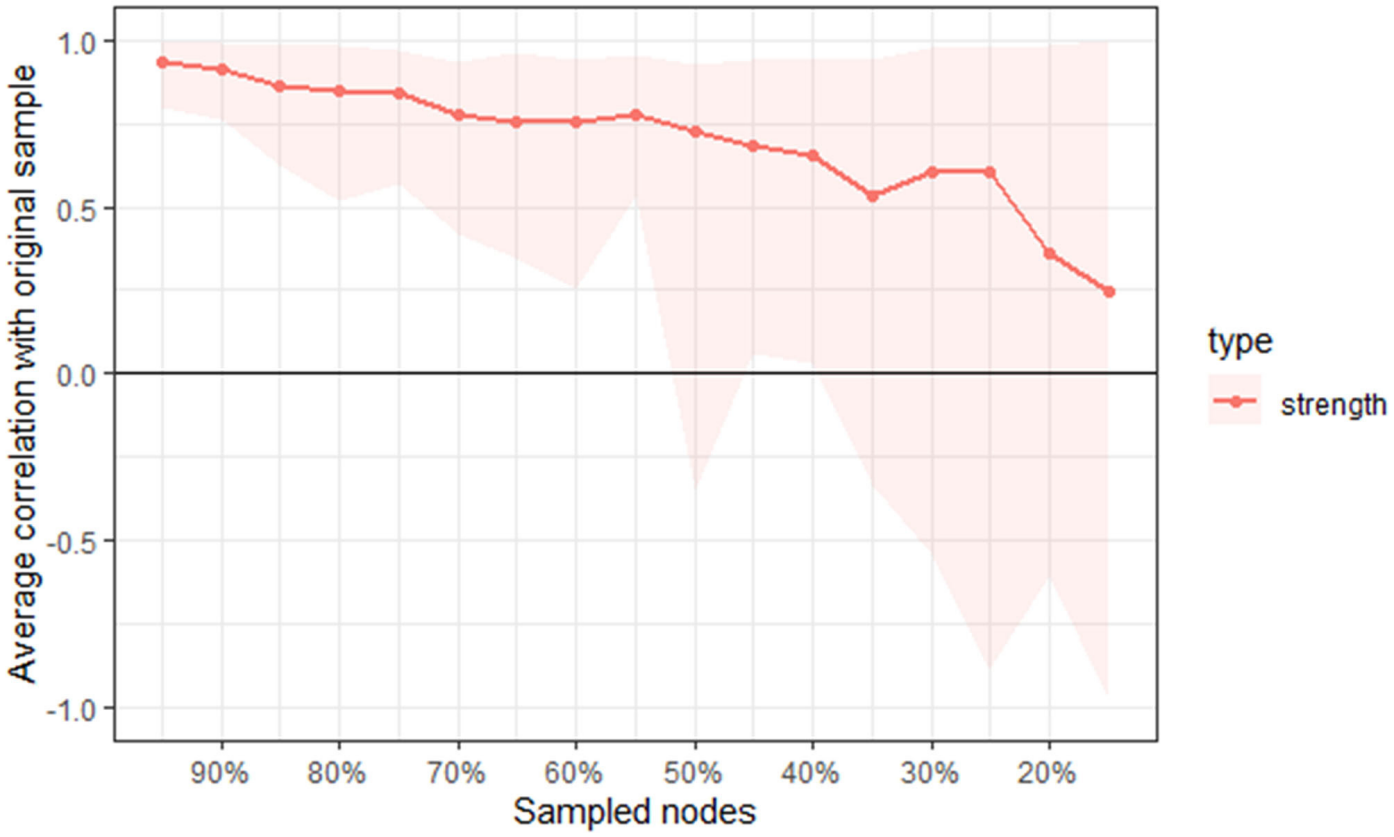
The average correlation between bootstrap centrality indices of networks sampled with node-dropping and network of the DSM-5 PTSD symptoms. PTSD, post-traumatic stress disorder; DSM-5, Diagnostic and Statistical Manual of Mental Disorders, Fifth Edition.

Figure 5 shows the estimated stability of the centrality indices for the 20 PTSD symptom network *via* node-dropping bootstrap technique. The results indicated a CS coefficient of 0.28 for strength.

## Discussion

The present study investigated the network of PTSD symptoms on people who were exposed to the COVID-19 outbreak. Specifically, we estimated and tested the accuracy and stability of two networks. One network contained 20 PTSD symptoms, and the other one included the 20 PTSD symptoms as well as six covariates. We will discuss the connections between the PTSD symptoms and the most central symptoms. We then discuss the relationships between the PTSD symptoms and the covariates.

### Post-traumatic Stress Disorder Symptoms

The results showed strong connections between avoidance of thoughts (C1) and avoidance of reminders (C2), between hypervigilance (E3) and exaggerated startle response (E4), between intrusive thoughts (B1) and nightmares (B2), between flashbacks (B3) and emotional cue reactivity (B4), and between detachment (D6) and restricted affect (D7) in the network of PTSD symptoms related to the COVID-19 pandemic. It suggested that the most central symptom was self-destructive/reckless behavior (E2).

In the 20 PTSD symptom network, the strong connections between hypervigilance and exaggerated startle response and between intrusive thoughts and nightmares were consistent with previous studies (16, 27, 33–35, 37). The strong connection between hypervigilance and exaggerated startle response indicated that the two symptoms affect each other through a feedback loop (16). It was supported by the Sensitization Model of PTSD. According to this model, survivors may become sensitive to the threat and show an exaggerated startle response after exposure to traumatic occurrences (60). Similarly, the strong connection between intrusive thoughts and nightmares indicated a loop in which intrusive thoughts about the traumatic event increase the possibility of nightmares associated with trauma, and in turn, the nightmares may make traumatic recollections more intrusive (34). In addition, the strong connection between detachment and restricted affect was also consistent with previous studies (16, 33, 35, 36, 38). This finding reflected that individuals with PTSD symptoms may regulate their emotions by disengaging from their emotions rather than engaging in the emotions. As a result, they may not only disengage from negative emotions related to trauma but also disengage from positive emotions after trauma (61, 62).

Trauma-related amnesia showed the lowest node strength in the network of DSM-5 PTSD symptoms. This finding was compatible with previous findings of PTSD network analysis (16, 33, 34, 37). Trauma-related amnesia has been suggested to be less useful in PTSD diagnosis (63, 64). Furthermore, trauma-related amnesia showed a very weak factor loading in confirmatory factor analysis (65). It seems that PTSD associated with vivid traumatic memories rather than trauma-related amnesia (66). These findings suggested that trauma-related amnesia might not be a central symptom of PTSD. Moreover, when we excluded “amnesia” from the network analysis, the structure was hardly influenced (see Supplementary Materials for more details).

The finding of strong connection between avoidance of thoughts and avoidance of reminders conflicted with previous studies in which there was no strong connection. This incongruence may be partly the result of different types of trauma (18). Similarly, network analysis for depression also found different connections among symptoms due to different life events (67, 68). The different intervals between traumatic event and conducting studies may also have contributed to this discrepancy. We conducted the investigation around 1 month after COVID-19 was controlled in China, while previous studies performed the studies much later after trauma than this present study (16, 33–35, 37).

Additionally, the strong connections among symptoms found in this study suggested that fear-conditioning models and dysphoric response might be central to the development of PTSD. That is, physiological and emotional responses to trauma cues and intrusive memories may lead to thoughts about traumatic events and avoidance of trauma cues (17), and intrusiveness and avoidance increase the sensitivity of perceived threats (35, 69), as suggested in the fear-conditioning models (35, 70, 71). Subsequently, increased sensitivity of threats eventually results in dysphoric responses such as hypervigilance and exaggerated startle responses (17). However, whether the development of PTSD symptoms in the context of the COVID-19 outbreak is compatible with these models or not still needs to be tested in longitudinal studies (69, 72).

In terms of the most central symptom, this study found that self-destructive/reckless behavior was at the center of the PTSD symptom network. The centrality analysis revealed that the strength of self-destructive/reckless behavior was significantly higher than that of other symptoms, while there was no significant difference of node strength between all the other symptoms. Therefore, self-destructive/reckless behavior might have the greatest clinical significance for the diagnosis of PTSD related to the COVID-19 pandemic. This symptom reflected high symptomatic burden and need for treatment. It is necessary to further investigate the factors that influence this symptom so as to develop more targeted interventions. However, this finding contrasted with most previous studies (16, 33, 35), which found self-destructive/reckless behavior to have only moderate centrality. This discrepancy may partly result from different types of trauma and different time points of investigating, as mentioned before. Additionally, different PTSD diagnostic criteria may also have contributed to this difference. For example, in some studies, the PTSD symptom networks were based on DSM-4 (16, 34, 35, 38), in which self-destructive/reckless behavior was not included as one of the PTSD symptoms.

The findings of the strongly connected symptoms and core symptom in this study have important implications for PTSD symptoms associated with the COVID-19 pandemic. The alleviation of these symptoms may benefit for reducing other symptoms (73–75). However, some studies failed to support this statement (76). A recent study found no difference between central symptoms and other symptoms in terms of their influences on symptom network (77). In addition, the centrality measurement is unable to reveal the direction of correlations between central nodes and other symptoms. Thus, some researchers suggested the most different symptoms as effective treatment targets (78). Moreover, the present study was conducted on healthy populations. Therefore, further longitudinal studies are needed to test directly on populations whether the identified strong connections and central symptom in this study can provide a viable treatment in psychotherapy.

### Post-traumatic Stress Disorder Symptom Network With Covariates

To extend the network of 20 PTSD symptoms, we included six clinically relevant covariates in the network. The strong connection between self-destructive/reckless behavior and suicidal ideation agreed with previous research, which revealed that self-destructive/reckless behaviors predicted suicidal ideation (33, 79). Moreover, PTSD itself was highly associated with suicidal thoughts (80). Self-destructive/reckless behavior may be a risk factor for suicide, and clinicians should pay more attention to trauma survivors with increased self-destructive behaviors. In addition, the results showed a strong association between loss of interest and depression. A recent study revealed that loss of interest was one of the hub symptoms within a network of PTSD and severe depression (81). The hub symptoms serve as bridges between disorders, increasing risk for comorbidity and severity of comorbidity. Additionally, it was unsurprising to find a strong connection among covariates between depression and anxiety symptoms. These two symptoms were frequently reported to be interrelated in previous network studies, and depression and anxiety are common comorbidities (82, 83). Therefore, it is necessary to consider depression and anxiety in the future studies of PTSD related to COVID-19.

In this present study, there was no impact of sex on the PTSD symptom network. This different finding from previous studies (36) might indicate that the impact of sex is on the overall connections of symptom network. Alternatively, this difference might due to our sample in which the number of females was much more than that of males. Interestingly, previous research has found that females were more vulnerable to PTSD than males (84, 85), while COVID-19-related studies found the opposite pattern (13, 15). Additional research that recruit equal female and male participants is necessary to investigate the effect of sex on the PTSD symptom network associated with COVID-19.

Consistent with previous studies, the PTSD symptom network has hardly changed when including covariates. It seems that the network of PTSD symptoms was relatively stable. However, it might also be due to the low scores of these variables in this study. More studies with larger samples are needed to test the effect of covariates on the PTSD symptom network.

In summary, the network analysis offers new insights into the interactions between PTSD symptoms themselves and other clinical conditions. The results had significant implications for understanding and intervention of PTSD related to the COVID-19 pandemic. Additionally, the sample set in this study included not only the participants who fulfilled the clinical diagnosis but also those who have not yet met clinical criteria. Previous studies have revealed that it is different between networks constructed based on clinical samples and non-clinical samples (86). Therefore, it is not enough to translate these findings into clinical practice. However, it is noteworthy that the individual difference of response to the COVID-19 outbreak is also clinically informative. COVID-19 is a threatening disease for human beings. It is unpredictable and need for distance and isolation. Moreover, the peri-traumatic phase of COVID-19 may be rather long (5). Therefore, it is important to help individuals who have a pathological burden but do not meet the PTSD diagnostic criteria to manage fears and worries and to develop coping skills for dealing with the ongoing threat.

### Limitations and Future Research Directions

Several limitations of this study need to be considered. First, this study collected cross-sectional data, which cannot identify causality between PTSD symptoms. As a result, it was not clear whether the most central symptom caused other symptoms or the other way around—or both. Therefore, future research that uses a longitudinal design is needed. Second, most of samples were college students (64.6%). They were under academic stress and exposed to relatively more social media, leading to serious vicarious trauma (87, 88). Furthermore, most of the samples were female. The findings in this study may be limitedly applied to young female populations. Therefore, these results require careful interpretation and translation into clinical practice. The robustness analyses revealed moderate instability, especially for the estimation of centrality parameters. The low stability of the network may be due to the small sample. Future studies with larger and sex-balanced samples are needed to improve the stability of the COVID-19-related PTSD symptom network. Third, the participants in this study were from different regions in China, where the severities of the COVID-19 pandemic were various. Consequently, the different severities of the COVID-19 outbreak may result in different symptoms and symptom networks. It is especially necessary to investigate the network of PTSD symptoms in the hard-hit regions by COVID-19 in the future. In addition, we have not checked if the participants had a PTSD history, which might interfere with the findings of PTSD symptom network (89). Fourth, this study used self-reported data, which limited objectivity and reliability (90). Future studies need to evaluate the PTSD symptom network more correctly through structured clinical interviews. It may be able to identify PTSD symptoms that are specific to the COVID-19 crisis. In addition, it is necessary to incorporate physiological and behavioral data to reveal the automatic processes that maintain PTSD symptoms in future research.

## Conclusion

The present study is, to our knowledge, the first to perform a network analysis of PTSD symptoms related to the COVID-19 outbreak. The results showed strong connections between avoidance of thoughts and avoidance of reminders, between hypervigilance and exaggerated startle response, between intrusive thoughts and nightmares, between flashbacks and emotional cue reactivity, and between detachment and restricted affect in the network of PTSD symptoms related to the COVID-19 pandemic. The most central symptom was self-destructive/reckless behavior. These results had significant implications for understanding and intervention of PTSD related to the COVID-19 pandemic. We emphasize the self-destructive/reckless behavior as an important target in the treatment of PTSD, which may facilitate relief of most PTSD symptoms.

## Data Availability Statement

All datasets generated for this study are included in the article/Supplementary Material.

## Ethics Statement

The studies involving human participants were reviewed and approved by the Ethic Institutional Review Board of Central China Normal University. The ethics committee waived the requirement of written informed consent for participation.

## Author Contributions

All authors reviewed drafts of the paper. WJ: performed the experiments, wrote-original draft, and prepared figures and tables. ZR: designed the experiments and project administration. LY: conceptualization, methodology, and designed the experiments. YT: wrote-review and editing. CS: contributed reagents, materials, analysis tools. All authors contributed to the article and approved the submitted version.

## Conflict of Interest

The authors declare that the research was conducted in the absence of any commercial or financial relationships that could be construed as a potential conflict of interest.
