# Supplementary material for: A Network Analysis of Post-traumatic Stress Disorder Symptoms and Correlates During the COVID-19 Pandemic
Source: Front Psychiatry. 2020 Nov 10;11:568037. doi: 10.3389/fpsyt.2020.568037 (PMC7683419; doi:10.3389/fpsyt.2020.568037)
Supplement: Supplementary file 1 [file Data_Sheet_1.docx]

**Supplementary Material**

**Data**

Covariance matrix

|  | B1 | B2 | B3 | B4 | B5 | C1 | C2 | D1 | D2 | D3 | D4 | D5 | D6 | D7 | E1 | E2 | E3 | E4 | E5 | E6 | Sex | Anx | Dep | SI | SS | SF |
| --- | --- | --- | --- | --- | --- | --- | --- | --- | --- | --- | --- | --- | --- | --- | --- | --- | --- | --- | --- | --- | --- | --- | --- | --- | --- | --- |
| B1 | 0.70 |  |  |  |  |  |  |  |  |  |  |  |  |  |  |  |  |  |  |  |  |  |  |  |  |  |
| B2 | 0.36 | 0.47 |  |  |  |  |  |  |  |  |  |  |  |  |  |  |  |  |  |  |  |  |  |  |  |  |
| B3 | 0.41 | 0.33 | 0.75 |  |  |  |  |  |  |  |  |  |  |  |  |  |  |  |  |  |  |  |  |  |  |  |
| B4 | 0.40 | 0.27 | 0.46 | 0.68 |  |  |  |  |  |  |  |  |  |  |  |  |  |  |  |  |  |  |  |  |  |  |
| B5 | 0.25 | 0.25 | 0.33 | 0.31 | 0.48 |  |  |  |  |  |  |  |  |  |  |  |  |  |  |  |  |  |  |  |  |  |
| C1 | 0.33 | 0.28 | 0.36 | 0.35 | 0.29 | 0.73 |  |  |  |  |  |  |  |  |  |  |  |  |  |  |  |  |  |  |  |  |
| C2 | 0.26 | 0.25 | 0.34 | 0.33 | 0.27 | 0.47 | 0.61 |  |  |  |  |  |  |  |  |  |  |  |  |  |  |  |  |  |  |  |
| D1 | 0.20 | 0.14 | 0.25 | 0.24 | 0.24 | 0.25 | 0.23 | 0.64 |  |  |  |  |  |  |  |  |  |  |  |  |  |  |  |  |  |  |
| D2 | 0.23 | 0.24 | 0.29 | 0.25 | 0.22 | 0.28 | 0.30 | 0.21 | 0.64 |  |  |  |  |  |  |  |  |  |  |  |  |  |  |  |  |  |
| D3 | 0.18 | 0.18 | 0.26 | 0.22 | 0.19 | 0.20 | 0.20 | 0.16 | 0.26 | 0.59 |  |  |  |  |  |  |  |  |  |  |  |  |  |  |  |  |
| D4 | 0.30 | 0.25 | 0.32 | 0.31 | 0.23 | 0.26 | 0.24 | 0.19 | 0.32 | 0.30 | 0.63 |  |  |  |  |  |  |  |  |  |  |  |  |  |  |  |
| D5 | 0.25 | 0.25 | 0.33 | 0.28 | 0.24 | 0.25 | 0.28 | 0.18 | 0.36 | 0.25 | 0.38 | 0.76 |  |  |  |  |  |  |  |  |  |  |  |  |  |  |
| D6 | 0.22 | 0.21 | 0.28 | 0.25 | 0.20 | 0.28 | 0.31 | 0.24 | 0.33 | 0.22 | 0.36 | 0.42 | 0.91 |  |  |  |  |  |  |  |  |  |  |  |  |  |
| D7 | 0.25 | 0.26 | 0.29 | 0.23 | 0.25 | 0.29 | 0.28 | 0.26 | 0.36 | 0.23 | 0.35 | 0.42 | 0.49 | 0.71 |  |  |  |  |  |  |  |  |  |  |  |  |
| E1 | 0.25 | 0.28 | 0.29 | 0.26 | 0.25 | 0.32 | 0.36 | 0.21 | 0.36 | 0.35 | 0.39 | 0.42 | 0.45 | 0.46 | 0.83 |  |  |  |  |  |  |  |  |  |  |  |
| E2 | 0.15 | 0.17 | 0.19 | 0.10 | 0.16 | 0.14 | 0.13 | 0.15 | 0.18 | 0.16 | 0.15 | 0.22 | 0.18 | 0.22 | 0.21 | 0.34 |  |  |  |  |  |  |  |  |  |  |
| E3 | 0.30 | 0.27 | 0.33 | 0.33 | 0.25 | 0.32 | 0.31 | 0.18 | 0.29 | 0.25 | 0.35 | 0.35 | 0.42 | 0.34 | 0.35 | 0.22 | 0.74 |  |  |  |  |  |  |  |  |  |
| E4 | 0.23 | 0.25 | 0.32 | 0.27 | 0.26 | 0.26 | 0.27 | 0.18 | 0.27 | 0.25 | 0.33 | 0.31 | 0.35 | 0.34 | 0.36 | 0.20 | 0.48 | 0.61 |  |  |  |  |  |  |  |  |
| E5 | 0.26 | 0.21 | 0.35 | 0.34 | 0.28 | 0.30 | 0.28 | 0.25 | 0.34 | 0.26 | 0.40 | 0.44 | 0.40 | 0.42 | 0.45 | 0.17 | 0.41 | 0.42 | 0.91 |  |  |  |  |  |  |  |
| E6 | 0.28 | 0.24 | 0.32 | 0.28 | 0.26 | 0.33 | 0.29 | 0.27 | 0.29 | 0.25 | 0.30 | 0.38 | 0.34 | 0.39 | 0.41 | 0.21 | 0.32 | 0.37 | 0.48 | 0.75 |  |  |  |  |  |  |
| Sex | -0.02 | -0.04 | -0.02 | 0.00 | -0.03 | -0.02 | -0.03 | -0.02 | -0.02 | -0.01 | -0.01 | 0.01 | -0.01 | -0.01 | -0.02 | -0.02 | -0.02 | 0.00 | 0.02 | 0.03 | 0.19 |  |  |  |  |  |
| Anx | 0.43 | 0.39 | 0.53 | 0.46 | 0.38 | 0.42 | 0.43 | 0.35 | 0.50 | 0.35 | 0.56 | 0.55 | 0.50 | 0.59 | 0.64 | 0.30 | 0.51 | 0.54 | 0.62 | 0.61 | -0.03 | 1.64 |  |  |  |  |
| Dep | 0.27 | 0.26 | 0.37 | 0.34 | 0.31 | 0.36 | 0.28 | 0.29 | 0.45 | 0.30 | 0.45 | 0.56 | 0.48 | 0.50 | 0.53 | 0.27 | 0.35 | 0.43 | 0.59 | 0.51 | 0.01 | 0.98 | 1.37 |  |  |  |
| SI | 0.15 | 0.20 | 0.23 | 0.09 | 0.22 | 0.19 | 0.16 | 0.18 | 0.25 | 0.19 | 0.22 | 0.28 | 0.21 | 0.30 | 0.22 | 0.34 | 0.25 | 0.25 | 0.22 | 0.25 | -0.05 | 0.46 | 0.45 | 0.78 |  |  |
| SS | -0.47 | -0.60 | -0.34 | -0.08 | -0.48 | -0.71 | -0.51 | -1.37 | -1.22 | -0.50 | -0.70 | -1.31 | -1.68 | -1.67 | -1.30 | -0.94 | -0.77 | -1.05 | -0.67 | -1.11 | 0.27 | -1.09 | -1.90 | -1.65 | 63.25 |  |
| SF | -1.37 | -1.16 | -2.00 | -1.74 | -1.57 | -1.67 | -1.41 | -1.44 | -2.15 | -1.56 | -2.18 | -2.34 | -2.27 | -2.39 | -2.68 | -1.02 | -1.62 | -1.85 | -2.71 | -2.23 | -0.04 | -4.23 | -3.92 | -1.95 | 15.98 | 37.30 |
|  |  |  |  |  |  |  |  |  |  |  |  |  |  |  |  |  |  |  |  |  |  |  |  |  |  |  |

Please use the Web view to see the full covariance matrix.

B1= Intrusive thoughts; B2 = Nightmares; B3 = Flashbacks; B4 = Emotional cue reactivity; B5 = Physiological cue reactivity; C1 = Avoidance of thoughts; C2 = Avoidance of reminders; D1 = Trauma-related amnesia; D2 = Negative belief; D3 = Blame of self or others; D4 = Negative trauma-related emotions; D5 = Loss of interest; D6 = Detachment; D7 = Restricted affect; E1 = Irritability; E2 = Self-destructive/reckless behavior; E3 = Hypervigilance; E4 = Exaggerated startle response; E5 = Difficulty concentrating; E6 = Sleep disturbance; Anx = Anxiety; Dep = Depression; SI = Suicidal ideation; SS = Social support; SF = Quality of life.

**Supplymentray Figure**


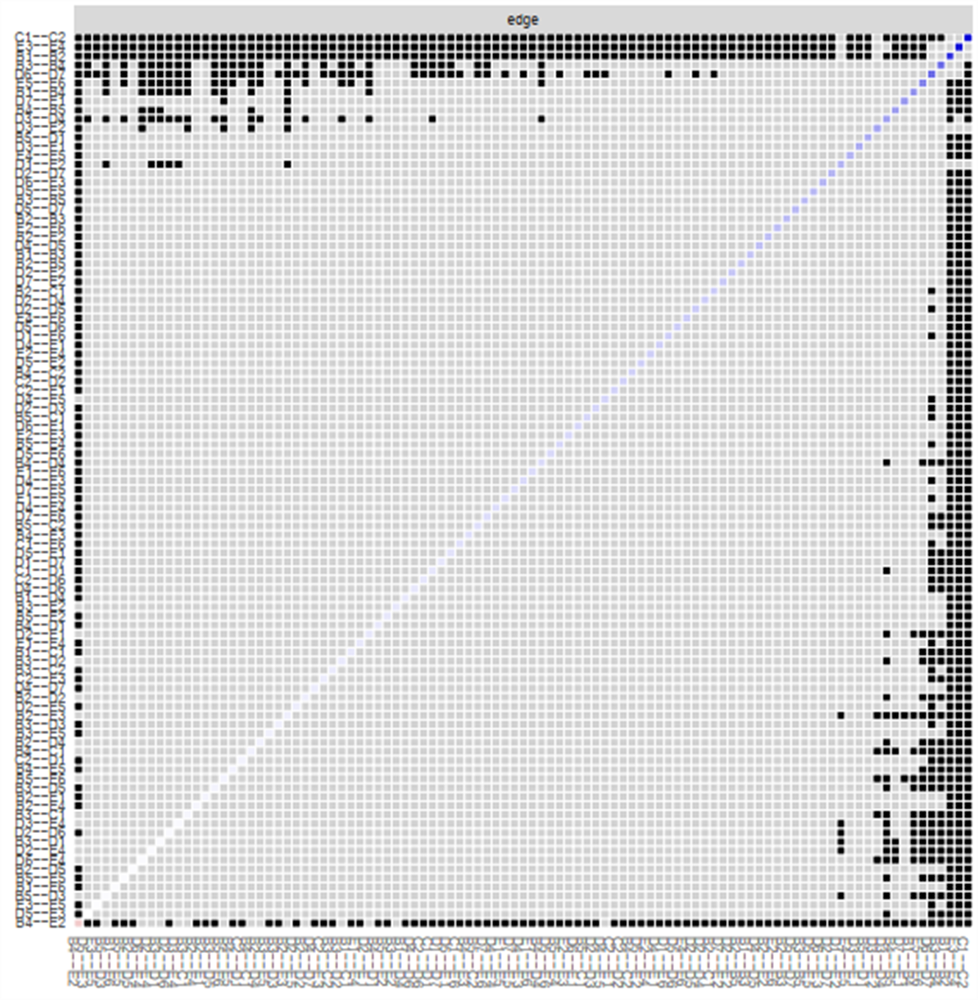


**Figure S1**. Bootstrap edge weights difference test between non-zero estimated edge weights in the network of 20 DSM-5 PTSD symptoms shown in Figure 1. Black boxes indicate a significant difference between two edges, gray indicate a non-significant difference. The color of the diagonal boxes (ranging from white to blue) corresponds to the thickness of the edge in Figure 1. B1 = Intrusive thoughts; B2 = Nightmares; B3 = Flashbacks; B4 = Emotional cue reactivity; B5 = Physiological cue reactivity; C1 = Avoidance of thoughts; C2 = Avoidance of reminders; D1 = Trauma-related amnesia; D2 = Negative belief; D3 = Blame of self or others; D4 = Negative trauma-related emotions; D5 = Loss of interest; D6 = Detachment; D7 = Restricted affect; E1 = Irritability; E2 = Self-destructive/reckless behavior; E3 = Hypervigilance; E4 = Exaggerated startle response; E5 = Difficulty concentrating; E6 = Sleep disturbance.


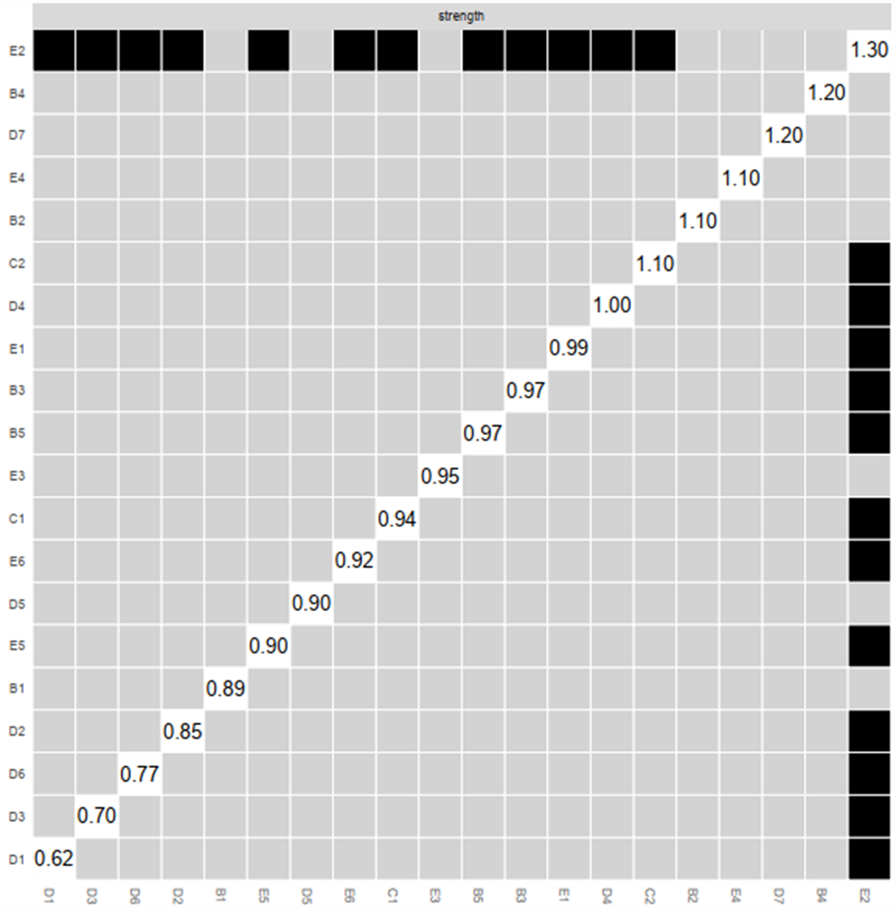


**Figure S2.** Bootstrap node strength difference test between node strength for the nodes of the network of 20 DSM-5 PTSD symptoms shown in Figure 1. Black boxes indicate a significant difference between two edges, gray indicate a non-significant difference. The number in the white boxes corresponds to the value of node strength. B1 = Intrusive thoughts; B2 = Nightmares; B3 = Flashbacks; B4 = Emotional cue reactivity; B5 = Physiological cue reactivity; C1 = Avoidance of thoughts; C2 = Avoidance of reminders; D1 = Trauma-related amnesia; D2 = Negative belief; D3 = Blame of self or others; D4 = Negative trauma-related emotions; D5 = Loss of interest; D6 = Detachment; D7 = Restricted affect; E1 = Irritability; E2 = Self-destructive/reckless behavior; E3 = Hypervigilance; E4 = Exaggerated startle response; E5 = Difficulty concentrating; E6 = Sleep disturbance.


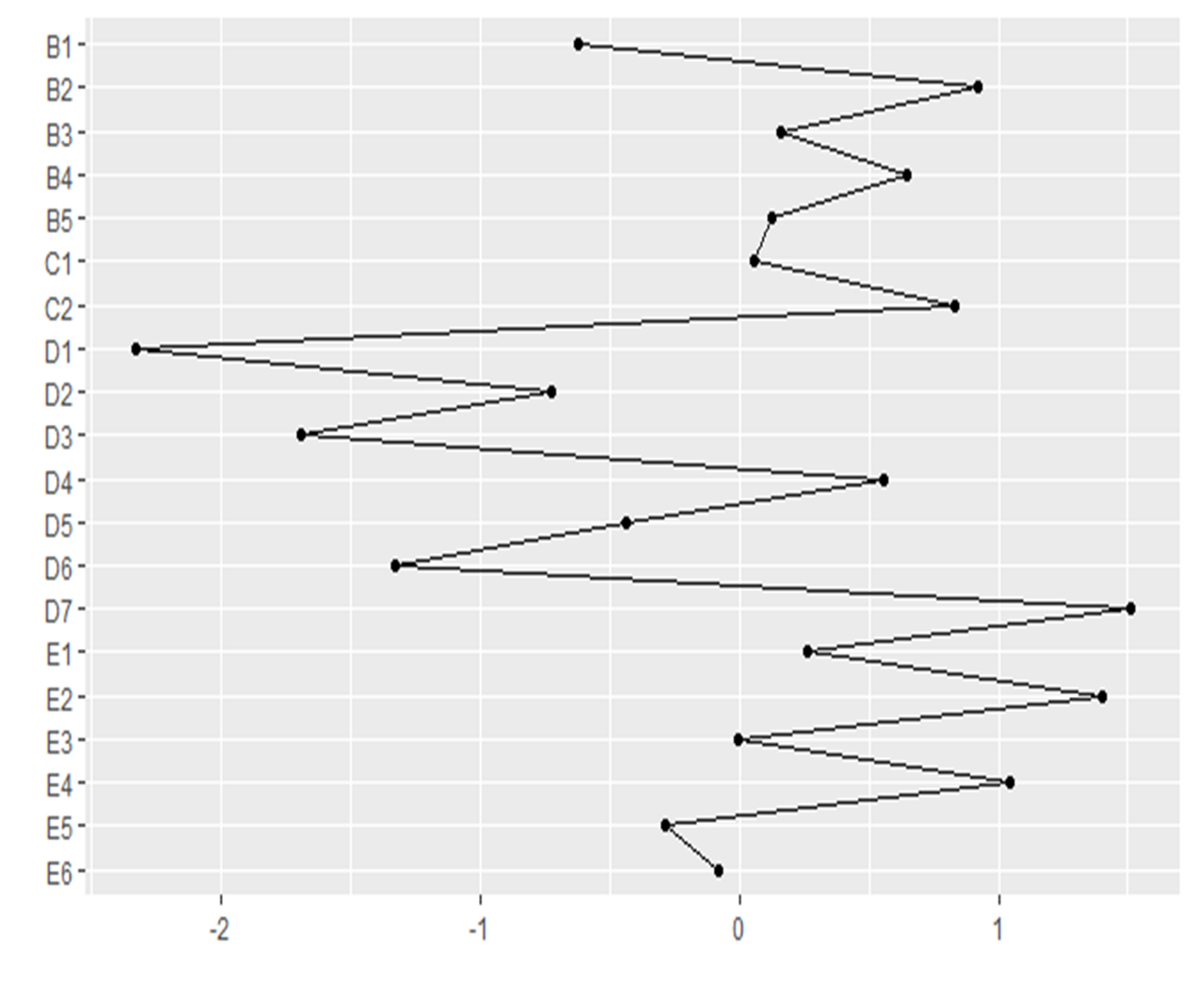


**Figure S3.** Node expected influence centrality for the estimated network of DSM-5 PTSD symptoms.

B1= Intrusive thoughts; B2 = Nightmares; B3 = Flashbacks; B4 = Emotional cue reactivity; B5 = Physiological cue reactivity; C1 = Avoidance of thoughts; C2 = Avoidance of reminders; D1 = Trauma-related amnesia; D2 = Negative belief; D3 = Blame of self or others; D4 = Negative trauma-related emotions; D5 = Loss of interest; D6 = Detachment; D7 = Restricted affect; E1 = Irritability; E2 = Self-destructive/reckless behavior; E3 = Hypervigilance; E4 = Exaggerated startle response; E5 = Difficulty concentrating; E6 = Sleep disturbance; PTSD = Posttraumatic stress disorder; DSM-5 = Diagnostic and Statistical Manual of Mental Disorders, Fifth Edition.


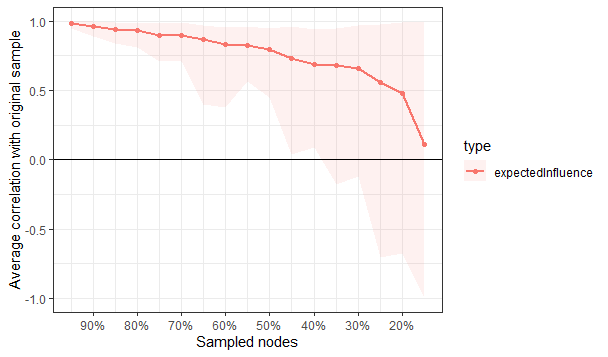


**Figure S4.** The average correlation between bootstrap expected influence of networks sampled with node-dropping and network of the DSM-5 PTSD symptoms. The CS coefficient of the expected influence is 0.44.


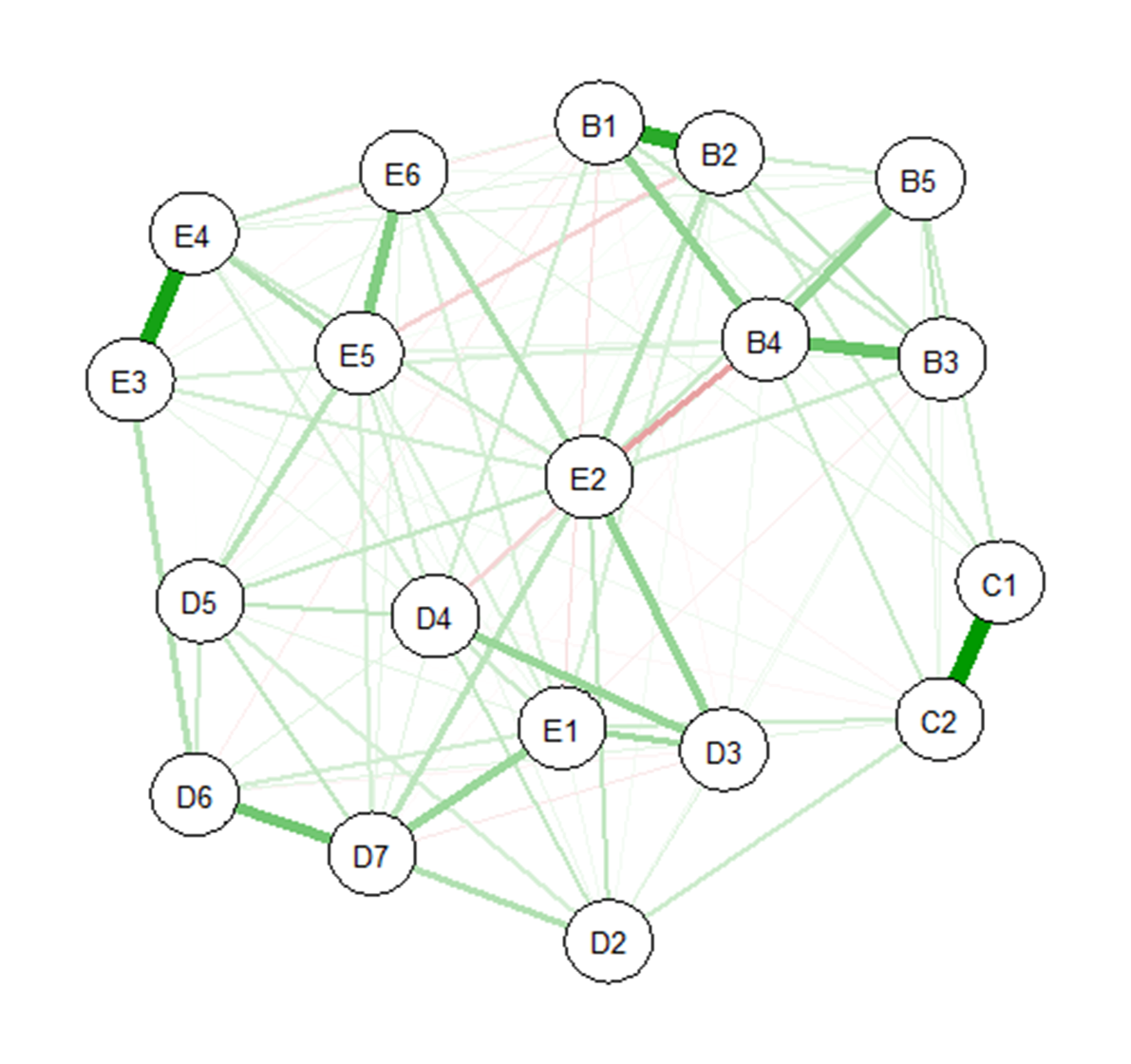


**Figure S5.** Estimated network of DSM-5 PTSD symptoms after removing the “trauma-related amnesia (D1)” item.

B1= Intrusive thoughts; B2 = Nightmares; B3 = Flashbacks; B4 = Emotional cue reactivity; B5 = Physiological cue reactivity; C1 = Avoidance of thoughts; C2 = Avoidance of reminders; D2 = Negative belief; D3 = Blame of self or others; D4 = Negative trauma-related emotions; D5 = Loss of interest; D6 = Detachment; D7 = Restricted affect; E1 = Irritability; E2 = Self-destructive/reckless behavior; E3 = Hypervigilance; E4 = Exaggerated startle response; E5 = Difficulty concentrating; E6 = Sleep disturbance.


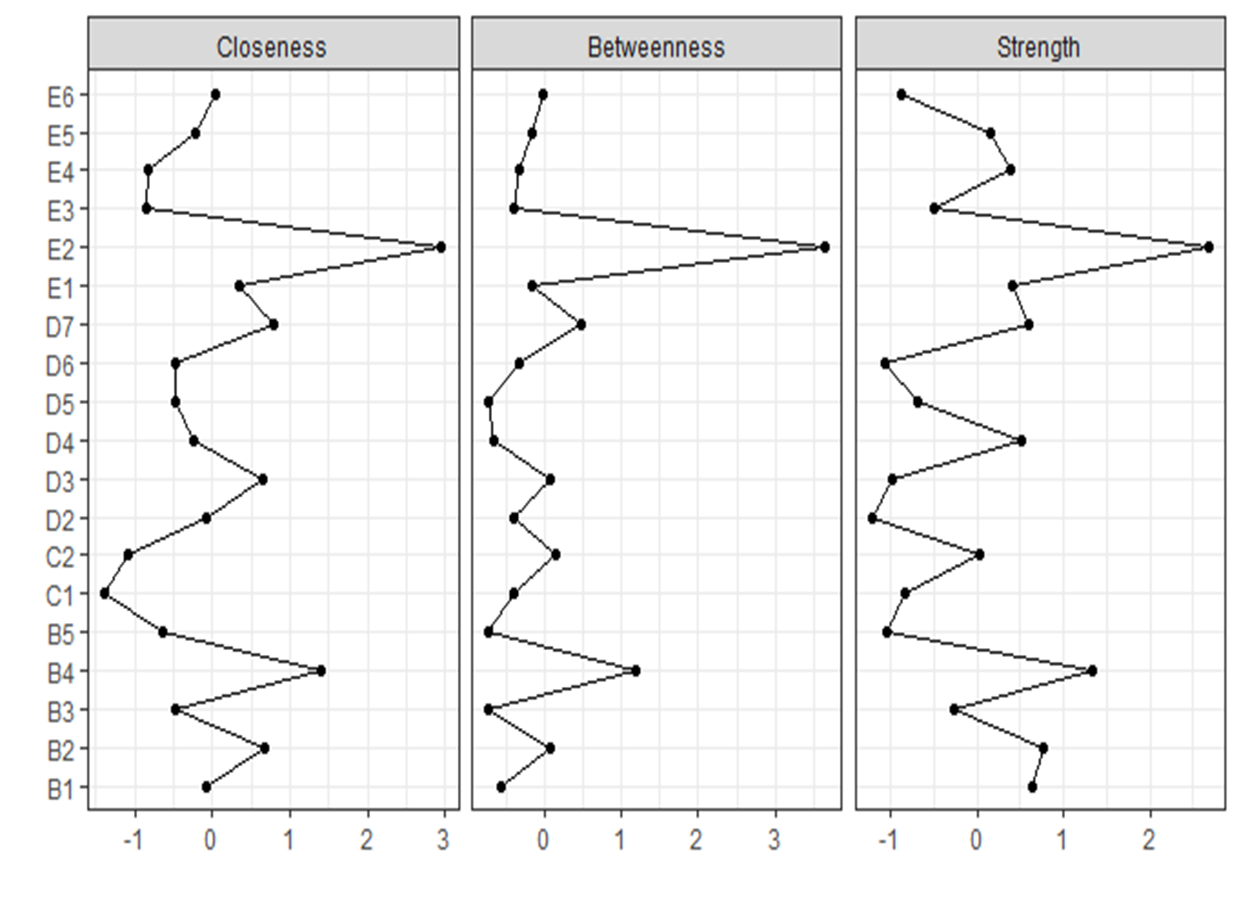


**Figure S6.** Closeness, betweenness, and node strength centrality indices for the estimated network of DSM-5 PTSD symptoms after removing the “trauma-related amnesia (D1)” item.


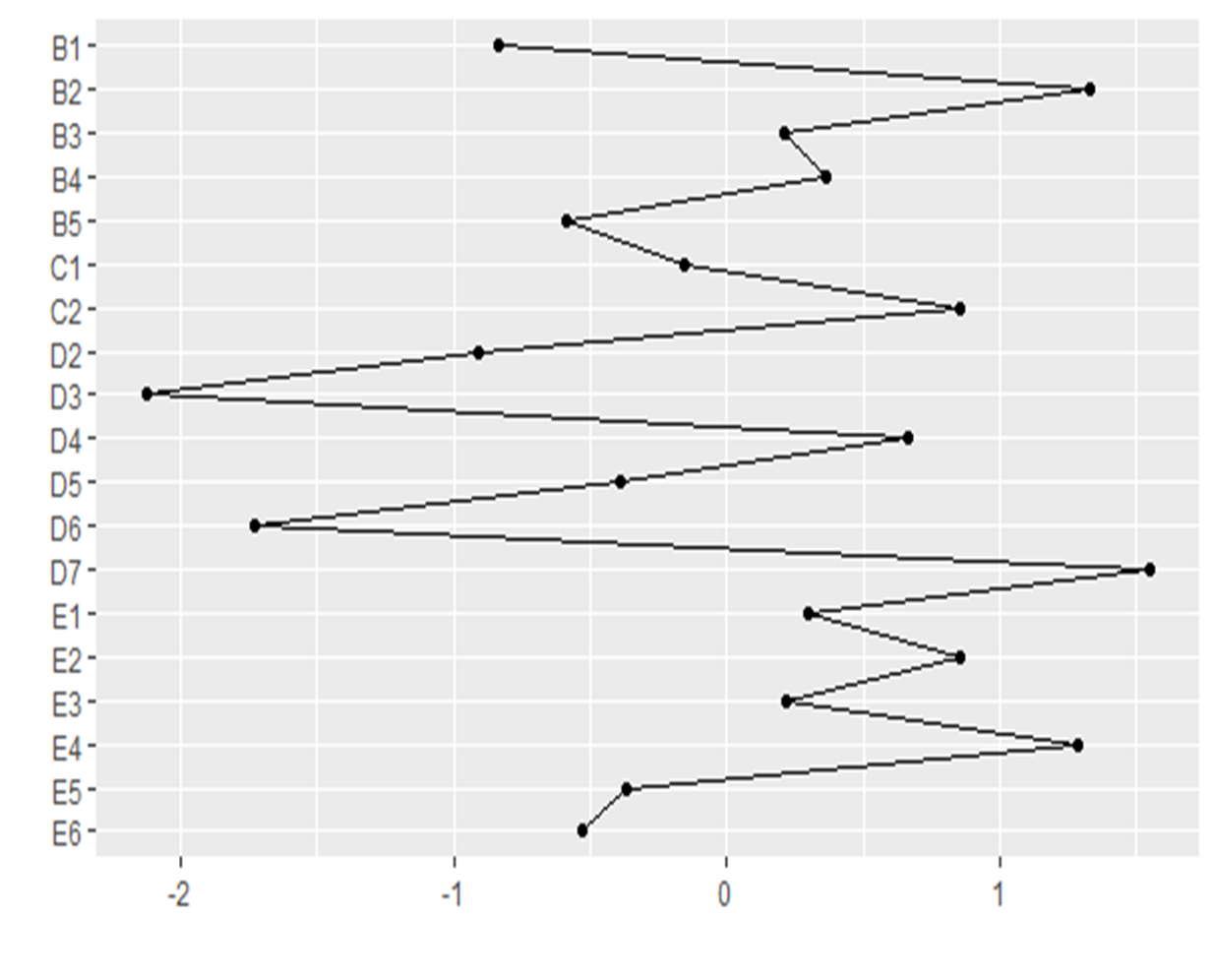


**Figure S7.** Node expected influence centrality for the estimated network of DSM-5 PTSD symptoms after removing the “trauma-related amnesia (D1)” item.


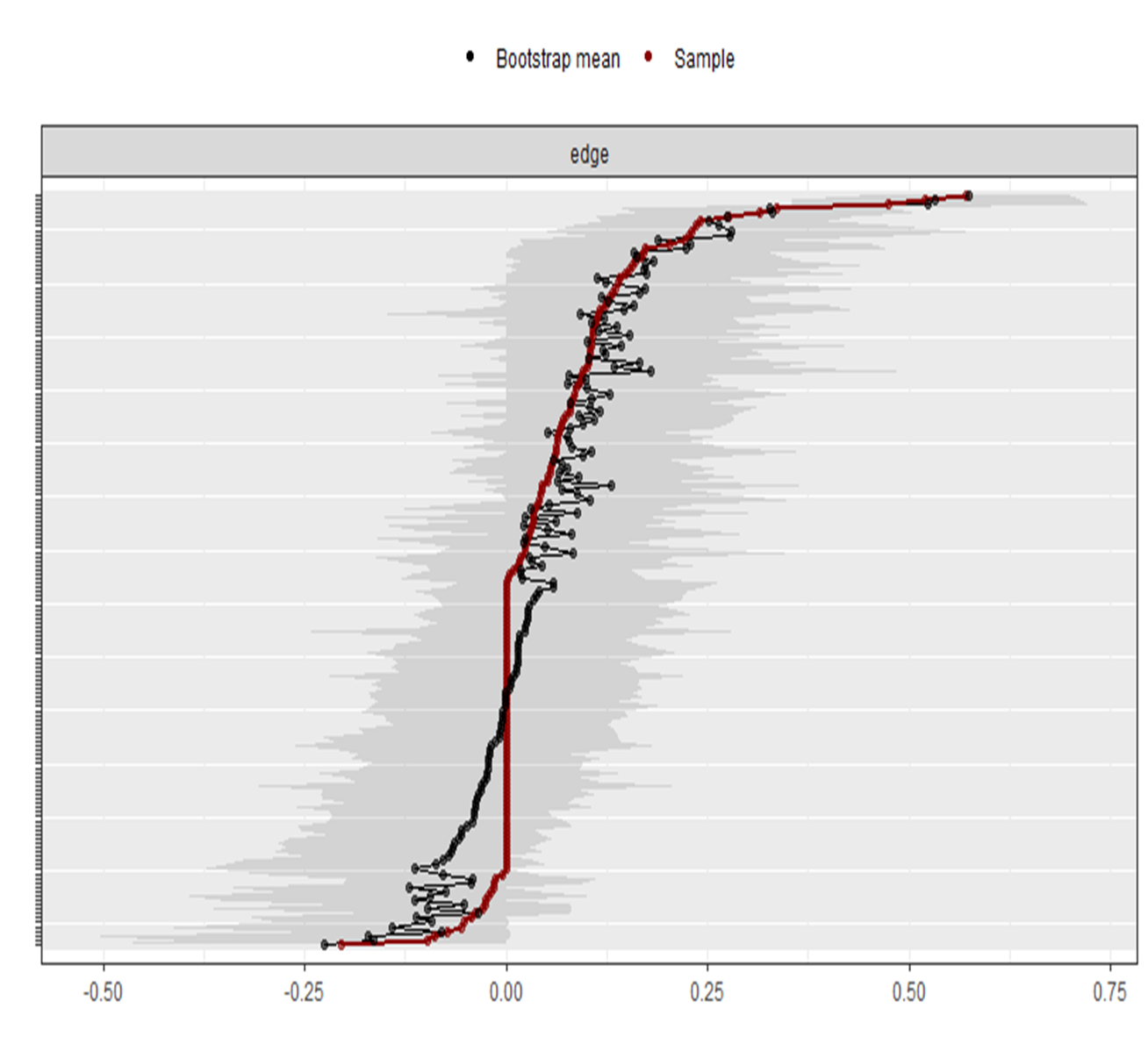


(a)


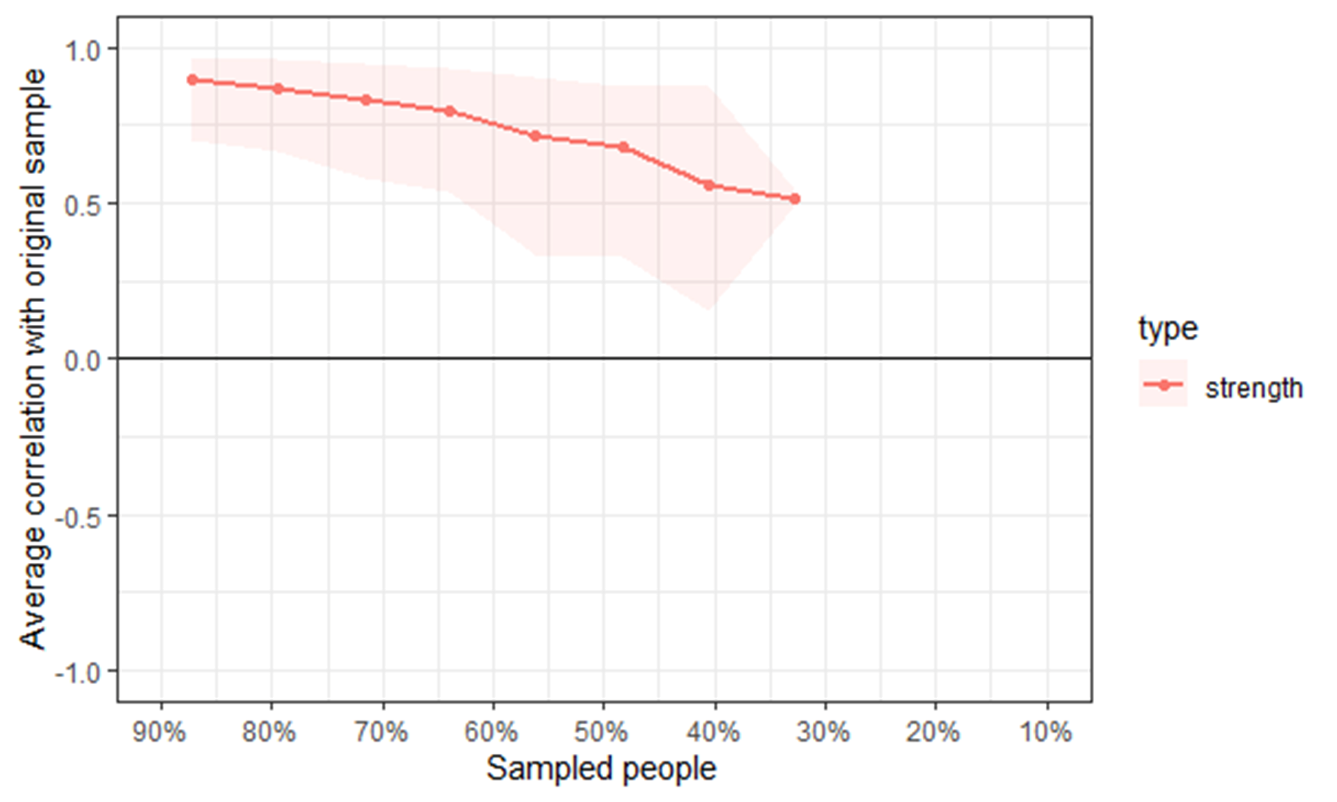


(b)


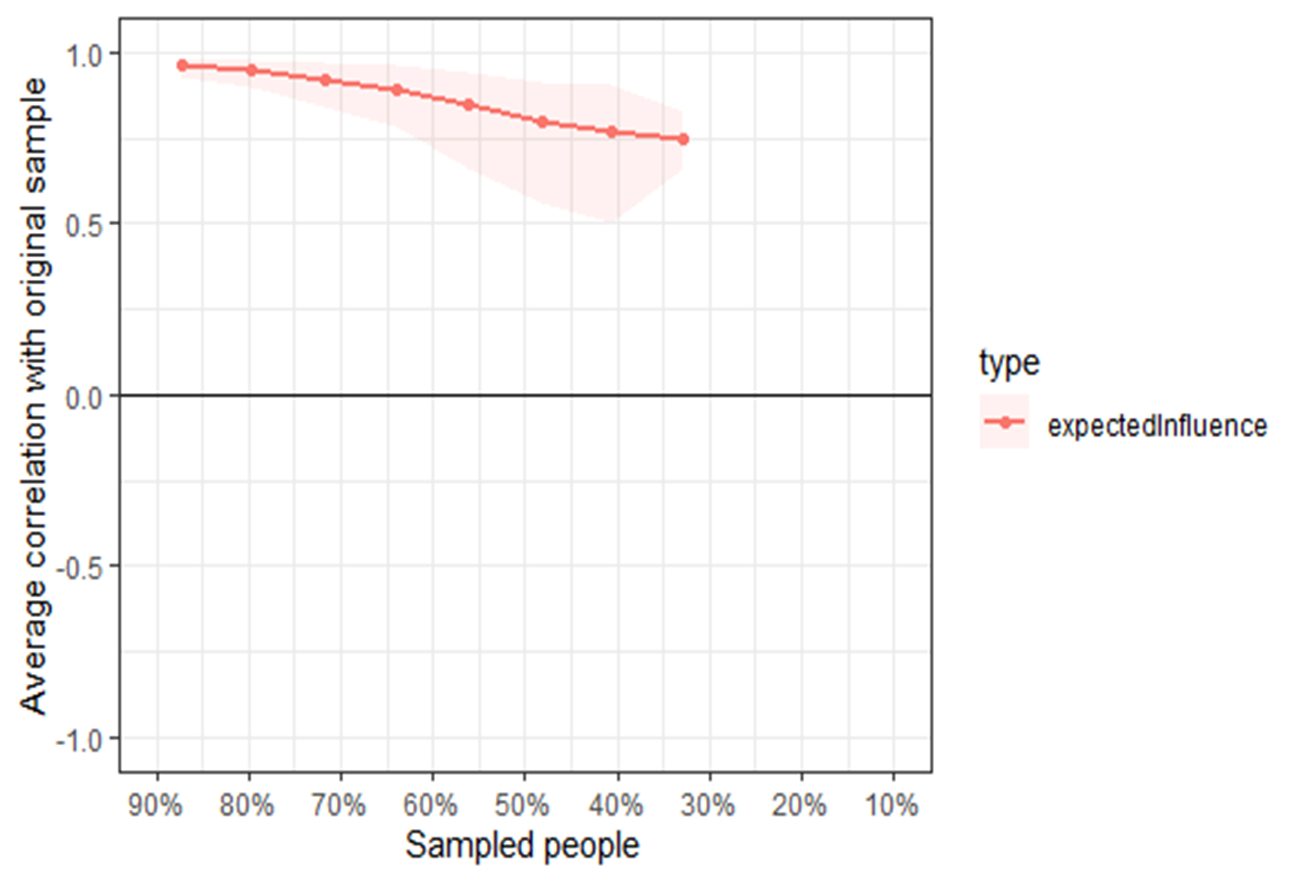


(c)

**Figure S8.** Robustness of networks. (a) Bootstrap 95% confidence intervals for estimated edge weights in DSM-5 PTSD symptom network after removing the “trauma-related amnesia (D1)” item. (b) The average correlation between bootstrap node strength of networks sampled with node-dropping and network of the DSM-5 PTSD symptoms after removing the “trauma-related amnesia (D1)” item. The CS coefficient of the node strength is 0.20. (c) The average correlation between bootstrap expected influence of networks sampled with node-dropping and network of the DSM-5 PTSD symptoms after removing the “trauma-related amnesia (D1)” item. The CS coefficient of the node strength is 0.36.


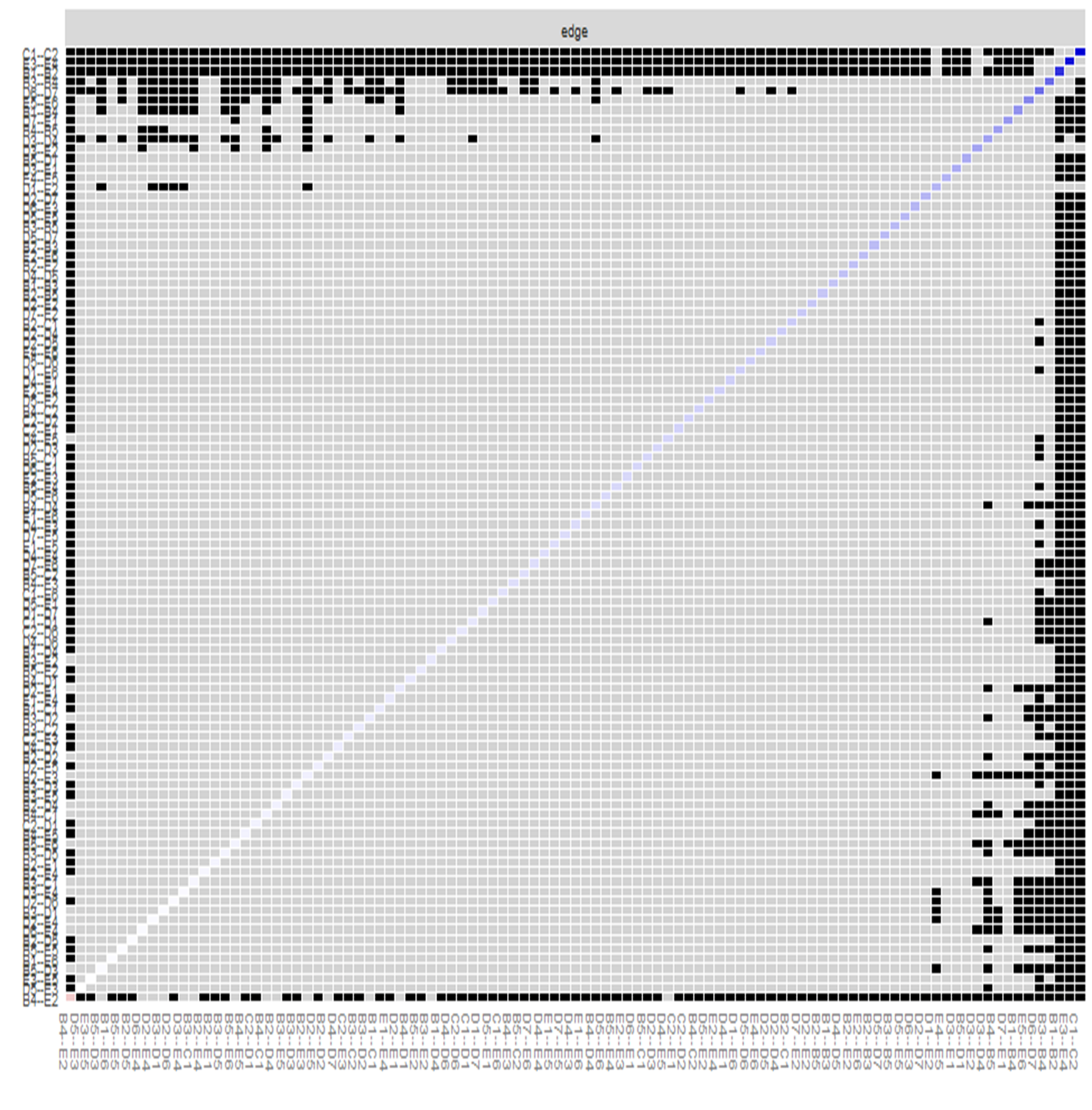


**Figure S9**. Bootstrap edge weights difference test between non-zero estimated edge weights in the DSM-5 PTSD symptom network after removing the “trauma-related amnesia (D1)” item shown in Figure S5. Black boxes indicate a significant difference between two edges, gray indicate a non-significant difference. The color of the diagonal boxes (ranging from white to blue) corresponds to the thickness of the edge in Figure S5.


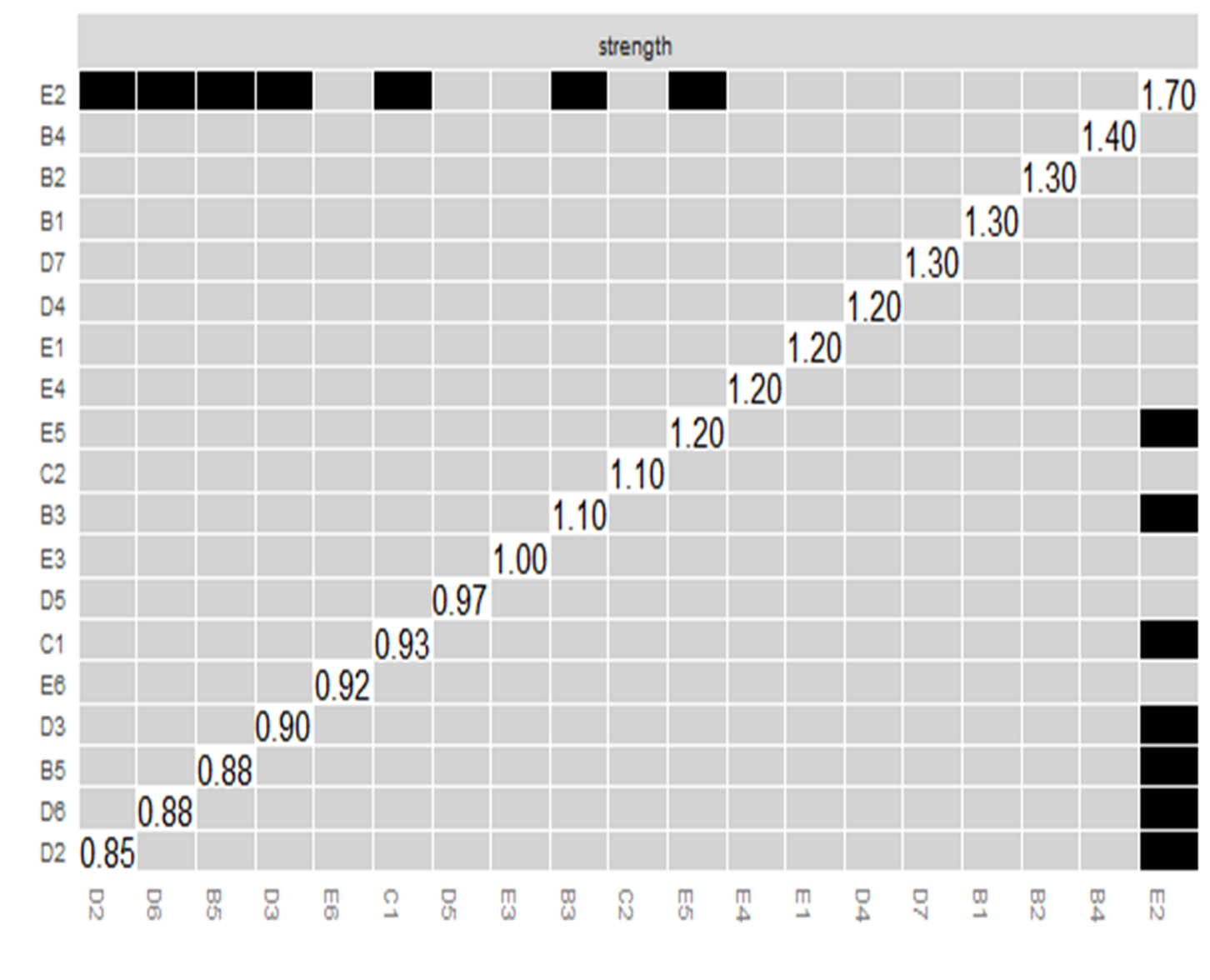


**Figure S10.** Bootstrap node strength difference test between node strength for the nodes of the network of DSM-5 PTSD symptoms after removing the “trauma-related amnesia (D1)” item shown in Figure S5. Black boxes indicate a significant difference between two edges, gray indicate a non-significant difference. The number in the white boxes corresponds to the value of node strength.

**R code used**

###Load libraries

library(qgraph)

library(bootnet)

library(networktools)

library(ggplot2)

**#### Part 1: PTSD symptom network**

**### Load data**

data1<-read.csv ("PTSD.csv",sep=",",na.strings = "99")

**### Estimation and visualization of network structure**

data1.cor<-cor_auto (data1)

graph1<-EBICglasso (data1.cor,n=nrow(data1), gamma = 0.5)

**### Centrality estimation**

**## Closeness，betweenness and strength**

qgraph::centralityPlot(graph1,include = c("Closeness","Betweenness","Strength"))

**## Node expected influence**

EI<-expectedInf(graph1)

EI$step1

plot(EI$step1)

**###** **Robustness estimation and testing for significance**

**## Bootstrapped CIs of edge weights**

network1<-estimateNetwork (data1, default = "EBICglasso",tuning=0.5)

boot1<-bootnet (network1, nBoots=1000, nCores = 8)

plot (boot1, order="sample", labels=FALSE)

**## Edge weight differences**

boot2<-plot(boot1, "edge", plot = "difference", onlyNonZero = TRUE, order = "sample", labels=TRUE)

plot(boot2, useDingbats=FALSE)

**## Node strength centrality differences**

boot3<-plot(bootSF, "strength", order="sample", labels=TRUE)

plot(boot3, useDingbats=FALSE)

**##** **Robustness estimation**

**# Node strength**

bootNODE<-bootnet(networkSF,nBoots = 1000,type="case",nCores = 8)

plot(bootNODE)

corStability(bootNODE)

**# Expected Influence**

bootEI<-bootnet(network1, statistics = "ExpectedInfluence", nBoots = 1000, type = "node")

plot(bootEI, statistics = "ExpectedInfluence")

corStability(booEI)

**#### Part2 PTSD symptom network with covariates**

**### Load data**

data2<-read.csv("CPTSD.csv", sep=",", na.strings = "99")

### **Estimation and visualization of network structure**

data2.cor<-cor_auto(data2)

graph2<-EBICglasso(data2.cor, n=nrow(data2), gamma = 0.5)

qgraph(graph2, layout="spring")

**### Robustness estimation**

network2<-estimateNetwork(data2,default = "EBICglasso",tuning=0.5)

bootCPTSD<-bootnet(network2,nBoots=1000,nCores=8)

plot(bootCPTSD, labels=FALSE, order="sample")

**#### Part3 PTSD symptom network after removing the ““trauma-related amnesia (D1)” item**

**### Load data**

data3<-read.csv("PTSD2.csv", sep=",", na.strings = "99")

### **Estimation and visualization of network structure**

data3.cor<-cor_auto(data3)

graph3<-EBICglasso(data3.cor, n=nrow(data3), gamma = 0.5)

**### Centrality estimation**

**## Closeness，betweenness and strength**

qgraph::centralityPlot(graph3,include = c("Closeness","Betweenness","Strength"))

**## Node expected influence**

EI2<-expectedInf(graph3)

EI2$step1

plot(EI2$step1)

**###** **Robustness estimation and testing for significance**

**## Bootstrapped CIs of edge weights**

network3<-estimateNetwork (data3, default = "EBICglasso", tuning=0.5)

boot4<-bootnet (network3, nBoots=1000, nCores = 8)

plot (boot4, order="sample", labels=FALSE)

**## Edge weight differences**

boot5<-plot(boot4, "edge", plot = "difference", onlyNonZero = TRUE, order = "sample", labels=TRUE)

plot(boot5, useDingbats=FALSE)

**## Node strength centrality differences**

boot6<-plot(boot4, "strength", order="sample", labels=TRUE)

plot(boot6, useDingbats=FALSE)

**##** **Robustness estimation**

**# Node strength**

bootNODE2<-bootnet(network3,nBoots = 1000,type="case", nCores = 8)

plot(bootNODE2)

corStability(bootNODE2)

**# Expected Influence**

bootEI2<-bootnet(network3, statistics = "ExpectedInfluence", nBoots = 1000, type = "node")

plot(bootEI2, statistics = "ExpectedInfluence")

corStability(booEI2)
